# Association between component-resolved diagnosis of house dust mite and efficacy of allergen immunotherapy in allergic rhinitis patients

**DOI:** 10.1186/s13601-019-0305-4

**Published:** 2019-12-19

**Authors:** Yanran Huang, Chengshuo Wang, Xi Lin, Hongfei Lou, Feifei Cao, Wenhan Li, Yuan Zhang, Luo Zhang

**Affiliations:** 1 Department of Otolaryngology Head and Neck Surgery, Beijing TongRen Hospital, Capital Medical University, Beijing, 100730 China; 2 Beijing Key Laboratory of Nasal Diseases, Beijing Institute of Otolaryngology, No. 17, HouGou HuTong, Dongcheng District, Beijing, 100005 China; 3 Department of Allergy, Beijing TongRen Hospital, Capital Medical University, Beijing, 100730 China; 4 EUROIMMUN Medical Diagnostics (China) Co., Ltd, Hangzhou, 310013 China; 5 Oumeng V Medical Laboratory, Hangzhou, 310013 China

**Keywords:** Allergic rhinitis, Allergen immunotherapy, House dust mite, Component resolved diagnosis

## Abstract

Data regarding clinical relevance of house dust mite (HDM) components over allergen immunotherapy (AIT) for allergic rhinitis (AR) are lacking. 18 adult AR patients receiving HDM-AIT for 52 weeks were followed up to assess serum levels of sIgE and sIgG4 to HDM components. The study showed that Der p1, p2, p23, Der f1 and f2, are important sensitizing components of HDM, of which Der p1 appears to be the most clinically relevant allergenic component for effective AIT.

To the editor,

Allergic rhinitis (AR) affects 10–40% of the global population and exerts huge economic and social burdens [1]. The most important allergen responsible for perennial AR (PAR) in China is HDM, including components *Dermatophagoides farina* (Der f) and *Dermatophagoides pteronyssinus* (Der p) [2].

Component resolved diagnostics (CRD) makes it possible for precision medication and individual management, while Der p 1, Der p 2, Der p 23, Der f 1 and Der f 2 are considered to be the main HDM allergens [3–5]. Allergen immunotherapy (AIT), including mainly subcutaneous immunotherapy (SCIT) and sublingual immunotherapy, is the only available etiological treatment for AR. Studies have shown that the levels of sIgE and sIgG4 actually increase during AIT [6, 7]. The levels of specific IgE present an early increase and a late decrease, while the levels of specific IgG4 show a relatively early increase instead [8]. Indeed, both sIgE and sIgG4 have been documented as potential biomarkers for monitoring clinical efficacy of AIT [9]. However, data regarding the clinical relevance of association between specific components of HDM and sIgE and sIgG4 levels during the course of AIT are still lacking. Thus, the aim of this study was to analyze the different HDM components and the profiles of sIgEs and sIgG4s generated against these components over a 1-year course of AIT in patients with HDM-induced AR.

The study was approved by the ethics review board of the Beijing TongRen Hospital (TRECKY2017-06). Study methods are described in Additional file 1 and a schematic diagram is provided in Additional file 1: Figure S1.

A total of 18 adult AR patients, receiving HDM-AIT for 52 weeks were serially followed up to assess serum levels of sIgE and sIgG4 to allergenic HDM components over the 52-week treatment. The demographic and clinical characteristics are shown in Additional file 1: Table S1. Clinically, symptoms were significantly improved during the 52-week HDM-AIT (detailed results were shown in Additional file 1).

Additional file 1: Table S2 shows the sensitization patterns of sIgE and sIgG4 to allergenic HDM components at baseline (W0), and indicate that the prevalence of sensitization to the individual components was in the order Der p 2 (66.7%) = Der f 2 (66.7%) > Der p 1 (55.6%) > Der f 1 (44.4%) > Der p 23 (33.3%); where sensitization to specific allergenic HDM components leading to generation of significant levels of sIgG was in the order Der f 2 (38.9%) > Der p 1 (27.8%) = Der p23 (27.8%) > Der p 2 (22.2%) = Der p 3 (22.2%). All participants were found to be negative (EAST class 0) with respect to significant generation of sIgE against Der p 3, Der p 5 and Der p 10; and generation of sIgG4 against Der p3, Der p10 and Der p23. Moreover, sIgE against Der p 7 and sIgG4 against Der p 7 and Der f 1 was found to be of little relevance, as they demonstrated intensities below 2 (clinically grade 0). However, 22% patients (4/18) were found to have clinically relevant sIgG4 levels against Der p 3.

The trends of the allergenic HDM components leading to production of sIgE are shown in Fig. 1. During the course of AIT, compared with W0 (baseline), the level of Der p sIgE was significantly elevated at W5 (25.27 ± 16.29 versus 20.13 ± 15.19, P = 0.022), and then decreased at W19 (15.53 ± 13.79, P = 0.047) before progressively attaining levels comparable with baseline at W52 (18.93 ± 16.04, P = 0.76). The production of Der p 1 and Der p 2 sIgE followed a trend consistent with Der p sIgE. In contrast, the levels of Der p 23 sIgE were significantly increased from baseline at W2 (25.61 ± 37.73 versus 18.56 ± 27.86, P = 0.027), and then remained significantly higher than the baseline throughout the rest of the treatment period (W5, 33.17 ± 43.64, P = 0.0078; W7, 30.67 ± 38.31, P = 0.025; W19, 28.89 ± 28.40, P = 0.028; W35, 30.29 ± 28.10, P = 0.049; W52, 37.11 ± 25.2, P = 0.0174).

**Fig. 1:**
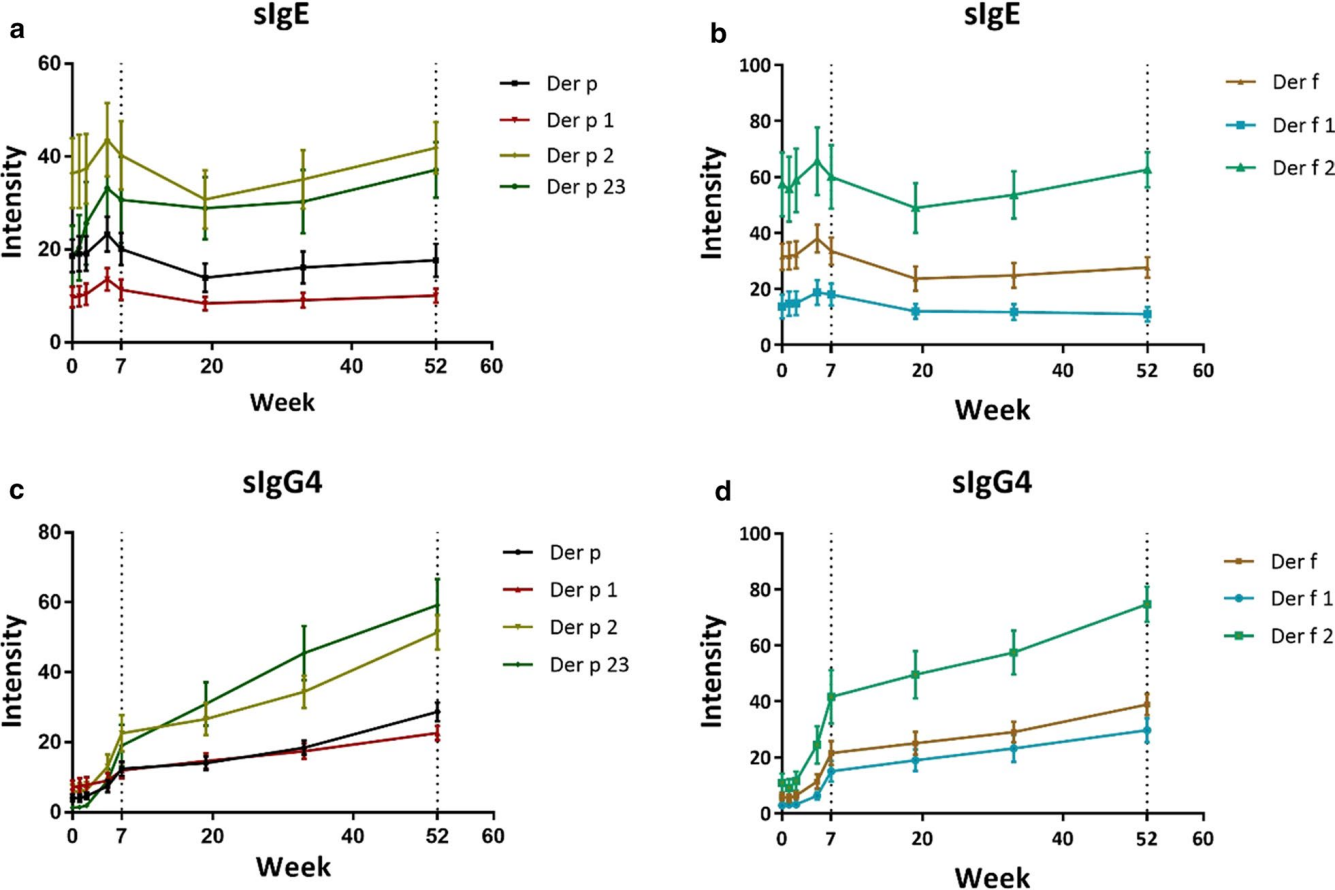
The profiles of sIgE and sIgG4 levels generated for allergenic HDM components during AIT. **a** sIgE of Der p and its components; **b** sIgE of Der f and its components; **c** sIgG4 of Der p and its components; **d** sIgG4 of Der f and its components. HDM, house dust mite; AIT, allergen immunotherapy

Similar to Der p sIgE, the level of Der f sIgE was also significantly elevated at W5 compared to baseline level (38.06 ± 20.77 versus 31.56 ± 19.94, P = 0.0049), and then decreased at W19 (23.67 ± 18.56, P = 0.020). Likewise, the levels of Der f 1 and Der f 2 sIgE significantly increased at W5 (Der f 1, 18.78 ± 18.87 versus 13.78 ± 18.05, P < 0.0001; Der f 2, 65.61 ± 51.3 versus 57.33 ± 48.23, P < 0.0001), but remained comparable at the other time-points.

The trends of the specific HDM components leading to production of sIgG4 are shown in Fig. 1. The levels of sIgG4 against Der p, Der f and their components (Der p 1, p 2, p 23, f 1 and f 2) were increased rapidly during the first 7 weeks of treatment and then progressively increased at a slower rate over the remaining course of the 52-weeks treatment.

The clinical efficacy of AIT was defined according the percentage improvement observed in total combined score (TCS) at 52 W compared with the baseline (W0) (with ≥ 20% improvement = responder; < 20% improvement = nonresponder) [10]. 6 out of 18 patients were unresponsive to HDM-SCIT and 12 patients responsive HDM-SCIT. Receiver operating characteristic curve analysis demonstrated that the ratio of sIgE and sIgG4 for Der p and Der p 1 were good predictors for clinical responsiveness, as demonstrated by areas under the curves (AUCs) of 0.75 and 0.92, respectively (Fig. 2a, b). Further, the Der p1 sIgE/sIgG4 was significantly and moderately correlated with the rate of clinical improvement in the patients (R = − 0.65, P = 0.0035; Fig. 2c).

**Fig. 2:**
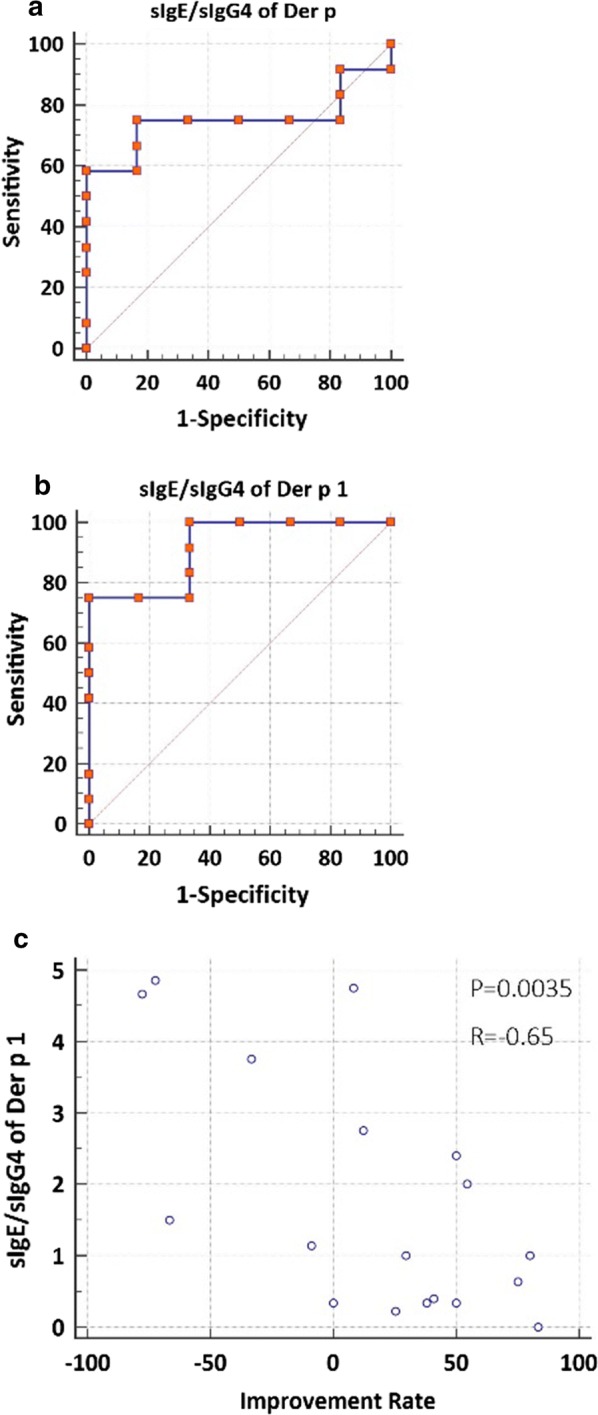
Receiver operating characteristic (ROC) curve of the baseline sIgE/sIgG4 ratio of Der p (**a**) and Der p 1 (**b**) as predictive parameters of clinical responsiveness, and the correlation between baseline sIgE/sIgG4 ratio for Der p 1 and clinical improvement rate for TCS at 52-weeks of AIT (**c**). AIT, allergen immunotherapy

A limitation of the study is that a 52-week course for AIT is relatively short compared to the traditional 3-year course for AIT. Secondly, the study population was relatively small.

In summary, this study has provided preliminary information of the allergenic profiles of major HDM components; especially Der p 1, Der p 2, Der p 23, Der f 1 and Der f 2; at baseline and over a course of 52-week AIT in patients with HDM-induced AR. Furthermore, Der p 1 appears to be the most clinically relevant allergenic component for effective AIT, and the ratio of Der p 1-sIgE/Der p 1-sIgG4 levels may be useful as a biomarker for predicting the clinical responses of AIT.

## Supplementary information

**Additional file 1.** Supplemental materials and methods. Demographic and clinical characteristics of the study population at baseline and clinical evaluation. **Table S1.** Demographics and baseline disease characteristics of participants. **Table S2.** Baseline levels of sIgE and sIgG4. **Figure S1.** Schematic diagram showing the SCIT protocol employed. **Figure S2.** The effect of HDM-AIT on nasal/conjunctivitis symptoms (A nasal congestion, B rhinorrhea, C nasal itching, D sneezing, E gritty eyes, F watery eyes) scores over a course of 52-weeks. AIT, allergen immunotherapy. **Figure S3.** The effect of HDM-AIT on DMS (A) and TCS (B) over a course of 52-weeks. DMS, daily medication score; TCS, total combined score; AIT, allergen immunotherapy.

## Data Availability

We would like to provide the raw data to support the information presented in this publication.

## Abbreviations

AR: allergic rhinitis
AIT: allergen immunotherapy
CRD: component resolved diagnostics
Der f: *Dermatophagoides farina*
Der p: *Dermatophagoides pteronyssinus*
HDM: house dust mite
PAR: perennial allergic rhinitis
SCIT: subcutaneous immunotherapy
sIgE: specific immunoglobulin E
sIgG4: specific immunoglobulin G4
TCS: total combined score
tIgE: total immunoglobulin E
WAO: World Allergy Organization

## References

[1] Brożek JL, Bousquet J, Agache I, Agarwal A, Bachert C, Bosnic-Anticevich S (2017). Allergic Rhinitis and its Impact on Asthma (ARIA) guidelines—2016 revision. J Allergy Clin Immunol.

[2] Lou H, Ma S, Zhao Y, Cao F, He F, Liu Z (2017). Sensitization patterns and minimum screening panels for aeroallergens in self-reported allergic rhinitis in China. Sci Rep.

[3] Zidarn M, Robic M, Krivec A, Silar M, Resch-Marat Y, Vrtala S (2019). Clinical and immunological differences between asymptomatic HDM-sensitized and HDM-allergic rhinitis patients. Clin Exp Allergy.

[4] Matricardi PM, Dramburg S, Potapova E, Skevaki C, Renz H (2019). Molecular diagnosis for allergen immunotherapy. J Allergy Clin Immunol.

[5] Matos Semedo F, Dorofeeva Y, Pires AP, Tomaz E, Taborda Barata L, Inacio F (2019). Der p 23-clinical relevance of molecular monosensitisation in House Dust Mite allergy. J Investig Allergol Clin Immunol.

[6] Zeng G, Zheng P, Luo W, Huang H, Wei N, Sun B (2016). Longitudinal profiles of serum specific IgE and IgG4 to Dermatophagoides pteronyssinus allergen and its major components during allergen immunotherapy in a cohort of southern Chinese children. Mol Immunol.

[7] Suarez-Fueyo A, Ramos T, Galan A, Jimeno L, Wurtzen PA, Marin A (2014). Grass tablet sublingual immunotherapy downregulates the TH2 cytokine response followed by regulatory T-cell generation. J Allergy Clin Immunol.

[8] Fujita H, Soyka MB, Akdis M, Akdis CA (2012). Mechanisms of allergen-specific immunotherapy. Clin Transl Allergy.

[9] Shamji MH, Kappen JH, Akdis M, Jensen-Jarolim E, Knol EF, Kleine-Tebbe J (2017). Biomarkers for monitoring clinical efficacy of allergen immunotherapy for allergic rhinoconjunctivitis and allergic asthma: an EAACI Position Paper. Allergy.

[10] Canonica GW, Baena-Cagnani CE, Bousquet J, Bousquet PJ, Lockey RF, Malling HJ (2007). Recommendations for standardization of clinical trials with Allergen Specific Immunotherapy for respiratory allergy. A statement of a World Allergy Organization (WAO) taskforce. Allergy.

