# Supplementary material for: Association between component-resolved diagnosis of house dust mite and efficacy of allergen immunotherapy in allergic rhinitis patients
Source: Clin Transl Allergy. 2019 Dec 19;9:64. doi: 10.1186/s13601-019-0305-4 (PMC6921530; doi:10.1186/s13601-019-0305-4)
Supplement: Supplementary file 1 — Additional file 1. Supplemental materials and methods. Demographic and clinical characteristics of the study population at baseline and clinical evaluation. Table S1. Demographics and baseline disease characteristics of participants. Table S2. Baseline levels of sIgE and sIgG4. Figure S1. Schematic diagram showing the SCIT protocol employed. Figure S2. The effect of HDM-AIT on nasal/conjunctivitis symptoms (A nasal congestion, B rhinorrhea, C nasal itching, D sneezing, E gritty eyes, F watery eyes) scores over a course of 52-weeks. AIT, allergen immunotherapy. Figure S3. The effect of HDM-AIT on DMS (A) and TCS (B) over a course of 52-weeks. DMS, daily medication score; TCS, total combined score; AIT, allergen immunotherapy. [file 13601_2019_305_MOESM1_ESM.docx]

**METHODS**

**Ethics**

This was a prospective cohort study, in which outpatients with HDM-induced AR were enrolled from the Department of Allergy at Beijing TongRen Hospital, from 2017 July to December. The study protocol, shown in Figure S1, was approved by the ethics review board of the Beijing TongRen Hospital and Beijing Institute of Otolaryngology, P.R. China; and written informed consent was obtained from all participants before enrolment into the study.

**Study Design**

Adult patients with mild to moderate AR, with or without asthma due to HDM, meeting the study inclusion criteria were enrolled into the study and received SCIT for 1 year with standardized extract of HDM (Alutard SQ, ALK company, Denmark). The 1-year clustered SCIT protocol involved a ‘7-week’ up-dosing (up to 100,000 SQ-U) and a ’45-week’ maintenance period with injection of allergen at intervals of 6 ± 2 weeks. The patients’ symptoms were evaluated at baseline and then followed up for a period of one year during the treatment. Blood samples from each patient were collected in 5-ml ethylene-diamine-tetraacetic acid (EDTA)-containing tubes at the initiation of treatment (W0), and the end of week (W)1, W2, W5, W7, W19, W35 and W52; and assessed for serum sIgE and sIgG4 to specific allergenic components of HDM; included Der p, p1, p2, p3, p5, p7, p10, p23, Der f, Der f 1 and f 2. All samples were analyzed using the semiquantified Euroline system (EUROIMMUN, Bejing, China), and the concentration the intensity and grade (EAST class) of each sample was graded as follows: intensity≤2= grade 0+; ≤6= 1+; ≤15, 2+; ≤30, 3+; ≤55, 4+; ≤110, 5+; ≤255, 6+.

**Patients**

A total of 18 outpatients, 10 males (55.6%) and 8 females (44.4%), aged 18 to 40 years with a confirmed diagnosis of AR were enrolled into the study. Each patient fulfilled the inclusion criteria as follows: (1) adult patients aged ≥ 18 years; (2) a history of atopy to HDM and a physician-diagnosis of mild to moderate rhinitis with or without asthma based on ARIA 2016 revision (1); and (3) patients with significant nasal symptoms (nasal congestion, rhinorrhea, itching and sneezing) and sensitization to HDM, as indicated by skin prick test (SPT) and confirmed by serum sIgE ≥ 3.5 kU/L using the Pharmacia UniCAP system (Thermo Fisher Scientific Co., Ltd., Shanghai, China). During AIT, patients only used rescue medication (antihistamines, nasal corticosteroids) if necessary.

Patients with severe uncontrolled systemic diseases, including active systemic/immunologic disease, were excluded; as were pregnant women.

**Clinical Efficacy of AIT**

The clinical efficacy of treatment was evaluated by assessing symptoms and rescue medication usage. Nasal symptoms of congestion, rhinorrhea, itching and sneezing, together with eye discomfort (gritty eyes and watery eyes) were assessed using a 4-point visual analogue scale (VAS) ranging from 0 (asymptomatic) to 3 (severe). Daily medication score (DMS), ranging from 0 to 6, was calculated based on the total daily medicine intake, with 0 = “without taking any medicine”, 1= “using antihistamines”, 2= “using nasal corticosteroids”, 3= “using oral corticosteroids”. Total combined score (TCS) (2), ranging from 0 to 6, was calculated as the sum of the average score of 6 nasal conjunctivitis symptoms and DMS.

According to the position paper of World Allergy Organization in 2007 (3), a ≥20% improvement in outcome is considered to be clinically efficacious for AIT. Thus, the clinical efficacy of AIT was defined according the percentage improvement observed in TCS at 52W compared with the baseline (W0) (with ≥20% improvement= responder; ＜20% improvement= nonresponder).

**Statistical analysis**

Data were analyzed using SPSS V.19 software package (IBM Corp., Armonk, NewYork, USA) and MedCalc V.15.2.2 (MedCalc Software, Mariakerke, Belgium). Descriptive statistics were used for general information of the study population. For normally distributed data the results were expressed as mean ± standard deviation (SD). Paired Student t test or Wilcoxon test were used to analyze the significance of differences in symptoms and medication scores between baseline and at different time points, for normally and non-normally distributed data. Receiver operating characteristic (ROC) curves were used to assess the predictive value of clinical responsiveness. Correlations were assessed using Spearman correlation. A value of P<0.05 was considered to be significant.

**RESULTS**

**Demographic and Clinical Characteristics of the Study Population at baseline**

The demographic and clinical characteristics of the study population are shown in Table S1. All 18 participants completed the 52-week SCIT treatment. The mean age of the patients was 30.17±9.67 years. Only 1 patient had history of asthma. There was no significant difference in patient number regarding gender. The baseline levels of sIgE to Der p and Der f were 18.61±14.85 and 31.56±19.94, respectively; and of sIgG4 to Der p and Der f were 4.11±4.42 and 5.94±5.79, respectively.

Der p and Der f were 4.11±4.42 and 5.94±5.79, respectively.

**Clinical evaluation**

Except for nasal congestion, the other 3 common nasal symptoms (rhinorrhea, itching and sneezing) and gritty eyes were significantly improved (0.78±0.73 versus 1.50±1.04, P=0.03; 1.22±1.00 versus 1.72±1.18, P=0.047; 1.06±0.80 versus 1.83±0.99, P=0.002; 0.83±0.71 versus 1.56±1.50, P=0.034; respectively) at the end of the 52-week SCIT treatment compared to the baseline (W0) (Figure.S2). Nasal congestion was found to significantly decrease at the W35 compared with W0 (0.94±0.83 versus 1.72±1.13, P=0.02). At W7, nasal itching and sneezing were shown to be the earliest effectively alleviated among the 6 nasal conjunctivitis symptoms (0.94±0.90 versus 1.72±1.18, P=0.027; 0.94±0.75, P=0.0078; respectively). However, no significant difference could be found in watery eyes (Figure S2F) and DMS during the 1-year treatment (Figure S3A). Combining symptoms and medication usage, TCS (Figure S3B) was found to significantly decrease at W35 and W52 compared with W0 (1.33±1.08, 1.50±0.99, respectively; versus 2.20±1.24 (W0); P=0.012 and 0.042, respectively).

**References**

1. Brożek JL, Bousquet J, Agache I, Agarwal A, Bachert C, Bosnic-Anticevich S, et al. Allergic Rhinitis and its Impact on Asthma (ARIA) guidelines—2016 revision. *Journal of Allergy & Clinical Immunology* 2017;**140**(4):950.

2. Baris S, Kiykim A, Ozen A, Tulunay A, Karakocaydiner E, Barlan IB. Vitamin D as an adjunct to subcutaneous allergen immunotherapy in asthmatic children sensitized to house dust mite. *Allergy* 2014;**69**(2):246–253.

3. Canonica GW, Baena-Cagnani CE, Bousquet J, Bousquet PJ, Lockey RF, Malling HJ, et al. Recommendations for standardization of clinical trials with Allergen Specific Immunotherapy for respiratory allergy. A statement of a World Allergy Organization (WAO) taskforce. *Allergy* 2007;**62**(3):317-324.

**TABLES**

**Table S1.** Demographics and baseline disease characteristics of participants

| Characteristics | Number |
| --- | --- |
| Total subjects, n | 18 |
| Gender (%), male/female | 10(55.6)/8(44.4) |
| Age (years; mean±SD) | 30.17±9.67 |
| Asthma (%), yes/no | 1(5.6)/17(94.4) |
| Baseline sIgE (mean±SD) |  |
| Der p | 18.61±14.85 |
| Der f | 31.56±19.94 |
| Baseline sIgG4 (mean±SD) |  |
| Der p | 4.11±4.42 |
| Der f | 5.94±5.79 |

**Table S2.** Baseline levels of sIgE and sIgG4

| Components of HDM | sIgE | | sIgG4 | |
| --- | --- | --- | --- | --- |
|  | Positive | Negative | Positive | Negative |
| Der p 1 | 10 (55.6%) | 8 (44.4%) | 5 (27.8%) | 13 (72.2%) |
| Der p 2 | 12 (66.7%) | 6 (33.3%) | 4 (22.2%) | 14 (77.8%) |
| Der p 3 | - | 18 (100%) | 4 (22.2%) | 14 (77.8%) |
| Der p 5 | - | 18 (100%) | - | 18 (100%) |
| Der p 7 | 1 (5.6%) | 17 (94.4%) | 1 (5.6%) | 17 (94.4%) |
| Der p 10 | - | 18 (100%) | - | 18 (100%) |
| Der p 23 | 6 (33.3%) | 12 (66.7%) | 5 (27.8%) | 13 (72.2%) |
| Der f 1 | 8 (44.4%) | 10 (55.6%) | 2 (11.1%) | 16 (88.9%) |
| Der f 2 | 12 (66.7%) | 6 (33.3%) | 7 (38.9%) | 11 (61.1%) |

**FIGURES**

**Figure legends**

Figure S1. Schematic diagram showing the SCIT protocol employed.

Figure S2. The effect of HDM-AIT on nasal/conjunctivitis symptoms (A nasal congestion, B rhinorrhea, C nasal itching, D sneezing, E gritty eyes, F watery eyes) scores over a course of 52-weeks. AIT, allergen immunotherapy.

Figure S3. The effect of HDM-AIT on DMS (A) and TCS (B) over a course of 52-weeks. DMS, daily medication score; TCS, total combined score. AIT, allergen immunotherapy.
